# Rendering white nationalism defeasible in interaction

**DOI:** 10.1111/bjso.70074

**Published:** 2026-04-24

**Authors:** J. Sterphone, Jessica Robles, Jack B. Joyce, Natalie Flint, M. J. Hill, Q. A. Ellis

**Affiliations:** ^1^ Department of Sociology Wheaton College Norton Massachusetts USA; ^2^ School of Social Sciences and Humanities Loughborough University Loughborough UK; ^3^ Nuffield Department of Primary Care Health Sciences University of Oxford Oxford UK; ^4^ Department of Sociology University of California Los Angeles Los Angeles California USA

**Keywords:** (in)sincerity, dog whistles, media interaction, membership categorisation analysis, racism, remediation, white nationalism

## Abstract

People work to present themselves as moral, reasonable or justified even when making racist or hateful comments. In this project, we identify interactional practices that accomplish support for White nationalism as part of a reasonable (or even positive) identity held by reasonable or positive actors. Using membership categorisation analysis and conversation analysis, we analysed a corpus of 24 publicly‐available video recordings for explicit mentions of (or challenges to) White nationalism/supremacy. Looking for how people explain, justify, and rationalise White nationalism and, especially, White nationalist violence, we identified 16 cases of what we call White nationalist remediation. Our findings demonstrate how not only self‐avowed White nationalists but also those who do not publicly identify as such can work to protect and potentially normalise White nationalist views and actions, including violence, using the same practices. They thus (1) signal their followers, (2) present their beliefs as reasonable and defensible, and (3) ultimately normalise White nationalism.

## INTRODUCTION

Political actors, among others, have exploited racial division for political gain without explicitly naming race as the matter‐at‐hand. This has been a recurrent political practice during both Trump administrations (2016–2020 and 2024–), amid anxieties concerning the rising prominence of the far‐right and perceived links between support of Trump and White nationalism, White supremacy and fascism (Kurylo & Reifowitz, 2025; Mirrlees, 2022). But these practices are not strictly new, having been profitable discursive resources in, for example, the Nixon administration (1969–1974), among others (see, e.g. Alexander, 2010; Nacos et al., 2024). Of course, these ideologies and practices cannot be localized only to the United States, as American influence mingles with local versions of White supremacy in other parts of the world (Wojczewski, 2024), and we find similar concerns prevalent in, for example, Germany (see, Sterphone, 2022a) and the UK (Parker, 2018).^1^

This work to invoke race without naming it often proposes (if implicitly) a race realist hierarchy of ‘real’ or ‘existent’ racial categories that have specific characteristics and participate in distinct kinds of activities (see, e.g. discussion in Whitehead et al., forthcoming). Additionally, they have proposed the (moral) superiority or legitimacy of one ‘race’, typically White people, such that they are more deserving of citizenship or other resources (i.e. racial [White] supremacy). One key resource for such talk has been the reciprocal relationship between categories and predicates/actions, which allows for talk about ‘inner city crime’, ‘council housing’, ‘crack cocaine’, etc. to be possibly hearable as talk about specific (racialised) categories of person. However, part of the power of such talk lies in its defeasibility, that is, its openness to revision towards another position that disclaims the relevance of race. Thus, speakers have, for example, advocated for policies that disproportionately target people of colour, spoken as advocates for racial hierarchy, and so on while having an out.

Increasingly, as race‐extremist political groups and positions enter mainstream political dialogue, members of society negotiate, in situ, whether and to what extent those groups and positions belong in the civil sphere (e.g. Sterphone, 2022a). Those holding these positions—whether as category members or not—have recurrently been shown to work towards remediating (Flint et al., 2019; Goffman, 1971) the morally problematised common‐sense knowledge about these positions and the categories affiliated with holding them, for example, White supremacist, Nazi, racist, White nationalist, member‐of‐the‐far‐right, etc. Remedial work, per Goffman (1971), serves to change the meaning of an act presently being treated as offensive or otherwise problematic into something more morally acceptable. For our purposes, actors may, in the process of providing remediating accounts as opposed to, say, apologies, work to validate and make ‘reasonable’ race realist and supremacist positions, as well as those that advocate violence (see below). This poses very real potential danger for the targets of such talk (racially minoritised persons, ‘the left’, etc.), and threatens the well‐being of society by propagating antagonism. Such talk is often subsumed under the broader category of *hate speech*. Hate speech generally refers to communicative acts—spoken, written, symbolic or behavioural—that are intended to denigrate, demean, or promote hostility towards a person or group on the basis of socially salient identity characteristics such as race, ethnicity, religion, or similar attributes (see Paz et al., 2020). Resultantly, such talk provides powerful incentive for scholars and members of society to understand how actors communicate these positions and negotiate their morality, particularly because ‘[a]s Sacks (1987: 67) noted, you cannot find what [people are] trying to do until you find the kinds of things they work with'—and if we wish to bring about change, we will need to know what we are up against’ (Rawls et al., 2020, p. 26).

Our focus is on what Garfinkel (1967) called the ethno‐methods, or members' (reasoning) practices for producing and navigating social order, through which members work to remediate a category like ‘White nationalist’ in situations where its morality becomes problematised, like by raising specific or category‐bound actions. In doing so, we demonstrate a recurrent orientation to a need to ‘remediate’ the morality of categories like ‘White nationalist’, ‘White supremacist’, and even ‘Nazi’. Across the two results sections, we demonstrate that this orientation is shared by actors who are self‐identified members of or participants in those categories, as well as those who are not, but may be situationally hearable as possible members. While each results section is concerned with one of the aforementioned categorial positions (member and non‐member), across them, we survey a set of practices used by *both* of these sets of persons, and take up dog whistling as one such practice deployed in the service of rehabilitation by categorial non‐members—those who might be situationally hearable as members, but are not explicitly categorizable as such. However, it is specifically in working to rehabilitate without being explicitly categorized as a member of the rehabilitated category—that is doing dog whistling—that they become situationally hearable as members.

## RACE, RACISM AND CATEGORIZATION

Social‐interactional and discursive psychological studies of race/racism centre demonstrable orientations to race and racism when looking for instances of race‐ and racist talk (see, Every & Agoustinos, 2007, p. 414; Schegloff, 2007b; Stokoe et al., 2025; Wetherell, 2003). Some might associate race talk and racism with explicit namings of racial categories or unambiguous statements of intentional racial hatred. But situated orientations to race (see discussions in Perkins et al., 2025; Wetherell & Potter, 1992) can be produced implicitly, covertly, and through the kinds of actions that are ‘common‐sensically’ (see, Garfinkel, 1967, p. 76; Whitehead et al., 2024) known to be done or had by certain types of person. These features of categories, called category‐bound actions and category‐tied predicates respectively (Sacks, 1992), emerge because categories operate as ‘the store house and filing system for common‐sense knowledge that ordinary people […] have about what people are like, how they behave, etc.’ (Schegloff, 2007b, p. 469). Because actions and predicates are for members recognizably linked to certain categories, they can be commutatively referenced *by* members: naming a category invokes the contextually relevant predicates and actions, and naming predicates and/or actions in the right context has the capacity to invoke a category (see, Whitehead et al., 2024).

The inference‐richness of categories enables the interactional study of race/racism beyond explicit namings of race/ism (e.g. Durrheim et al., 2015; Potter & Wetherell, 1987; Tileaga, 2005), which is essential given the apparent and pervasive cultural norm against ‘prejudice’ (Billig, 1988, p. 94). Members of society work to not be hearable as prejudiced or racist (or other ‐ists) (see, Billig, 1988; Goodman, 2014; Goodman & Johnson, 2013; Goodman & Rowe, 2013; Whitehead, 2009; Whitehead, 2020; Whitehead & Stokoe, 2015). Indeed, they deploy a variety of practices for circumventing being hearable as engaging in prejudicial talk—and thus as categorial racists, White supremacists, etc.—like, for example using seemingly innocent referents and symbols; avoiding racial categories altogether, perhaps in favour of naming category‐bound actions; setting up a puzzle and relying on the hearer to provide the racial‐category as the solution (Whitehead, 2009, p. 338); or topicalizing of friends or relations (Joyce & Sterphone, 2022). Such practices, along with others like disclaimers (Billig, 1988; Billig et al., 1988, p. 112; Whitehead, 2013), demonstrate both a speaker's orientation to the norm against prejudice, and its relevance to and consequentiality for their ensuing or preceding talk.

Speakers can both display an orientation to this norm and still produce hearably prejudicial speech by producing it as defeasible—that is, as specifically revisible and deniable (in third position) as having been prejudicial. Such talk is typically open‐to‐interpretation and hearable in more‐than‐one way, such that (self‐ or other‐initiated) repair provides opportunity to offer alternative, non‐prejudicial formulations (see, e.g. Robles, 2015; Weatherall, 2015). Designing one's hearably prejudicial talk defeasibly but as nonetheless hearably prejudicial, especially to those who are familiar with its features, are constitutive characteristics of what scholars have called ‘dog whistling’ (Haney Lopez, 2015, pp. 4–5, 130). Dog whistles are a form of coded (racial) appeal that rely on a relationship between what is overtly said and what is covertly suggested, or, as Haney Lopez (2015) puts it, ‘inaudible and easily denied in one range, yet stimulating strong reactions in another’ (3). There has been a resurgence in scholarly attention to the practice following the election to Donald Trump to the US presidency in 2016, with particular attention paid to moderated online spaces (see, e.g. Bhat & Klein, 2020; Lonsdale, 2021; Quaranto, 2022; Ray & Durham, 2020; Thompson & Busby, 2023).

One practice for managing deniability comes in designing talk in ways that can be heard as race‐neutral (Whitehead, 2020), which can provide for the possibility of problematizing others for hearing the relevance of race in their talk. We can find further evidence of orientations to norms around ‘race‐neutrality’ or ‘colour‐blindness’ (Bonila‐Silva, 2017; Frankenberg, 1993) in such practices, both in productions of non‐racialised talk and in counters (turning‐it‐around) that call complainants the ‘real racists’. Analysis of German‐language social media data following the Hanau White nationalist shootings similarly shows how racialised violence was reframed through suppressive racism‐denying formulations (Windel et al., 2023). The intersections of the covertness of talk, defeasibility, and counter accusations demonstrate the intricate complexities at the heart of managing the moral implications around producing and responding to racial/racist talk (see Whitehead, 2019). Indeed, even when racist comments are relatively explicit, participants work to present themselves within frameworks of morality or reasonableness (see Josey, 2010).

These forms of ‘coded talk’ notwithstanding, members of society demonstrably attend to and manage the socially available morality of categories of which they may be seen to be members (e.g. Hofstetter & Robles, 2019; Sterphone, 2022b; Whitehead, 2020), including in response to attributions of racism (e.g. Robles, 2015) or due to associations across categories (for example, the association between White nationalists and racism). Some practices for recovering the category and its moral standing include partitioning (Sacks, 1992) away from others who might be seen as more ‘problematic’ members *or* from others in the broader collection that the category exists within (similar to work on in‐ and out‐groups, c.f., Tajfel & Turner, 2004). Partitioning categories involves, divid[ing] themselves into different categories […] to belong to different ‘territories of ownership’ (Raymond & Heritage, 2006) and leverage the associated domains of knowledge and responsibilities tied to those identities (Joyce & Walz, 2022, p. 566, emphasis original); for example, separating being against immigration or asylum‐seekers from being racist (Goodman & Burke, 2010). This relies, in part, on notions of co‐ and cross‐membership, the latter referring to how members can be juxtaposed into oppositional categories from within the same ‘device’ to versus, for example lending legitimacy to a claim/stance (Sacks, 1992: 590; see, Whitehead & Lerner, 2022). These practices can be used to separate categories that may be seen as ‘tainted’ in some way from the person and their identification with some part of that category or associated category. This form of remediation or rehabilitation is part of the suite of resources participants have at their disposal when communicating interpretably racist views and seeking to avoid or recover from attributions of racism.

Research on the denial and management of prejudice in social interaction has focused largely on speakers' denials, disclaimers, and avoiding accountability, underscoring the centrality of moral reasoning in talk about race. We extend this work using conversation analysis and membership categorisation analysis to show how interlocutors can use the interactional machinery of categorisation and defeasibility not merely to deny racism, but to reconstitute the moral order of categories like ‘White nationalist’ or ‘far‐right’. In doing so, they work to rehabilitate possibly stigmatized identities, rendering them intelligible and reasonable.

## MATERIALS AND METHODS

This study tracks and scrutinizes interactional practices that rehabilitate White nationalism. These practices empower, garner support, and provoke opposition (Bhat & Klein, 2020). By analysing these practices using conversation analysis (CA) and membership categorisation analysis (MCA), the analysis investigates how participants attribute, disclaim, and challenge racism within naturally occurring interaction. The following section details the data and analytic procedures used to identify these practices.

### Data collection

Recordings collected for other projects (see Joyce, 2019; Joyce & Sterphone, 2022) were taken from multiple interactional contexts/genres including TV/Radio interviews and talk shows, publicly recorded dispute videos, news programmes, and protest recordings in two English‐speaking contexts (United States and UK). All data in this paper's analysis are from 2017 to 2023, in the United States and the UK (one case in the broader collection comes from Germany), which could seem a very broad context from which to draw. However, this time period was notably characterized by increased national and international attention to race and racism, as exemplified by the Black Lives Matter movement and consequences of the election of US president Trump, ‘Brexit’ (the UK leaving the European Union) vote, the so‐called European ‘migration crisis’, and the worldwide COVID pandemic. While we did not gather the data using this cultural background as part of our criteria, nor draw on it explicitly as part of our analysis, we acknowledge it is an important context that may highlight why different orientations to White nationalism emerge across such varied data. These recordings often comprise or arose in response to some notable politicized event or context within which ‘race’ was topicalized as a relevant matter. Our collection comprises 24 recordings (5 h and 17 min); a summary can be found in Table A1 (see Appendix A).

The original 24 recordings were reviewed to identify instances of explicit mention of White nationalism/supremacy or challenges to a participant's views when White nationalism was procedurally relevant (see Schegloff, 2007b). White nationalistic views were attributed on the basis of how participants themselves managed White nationalism as a self‐identification *or* as an attribution, or potential attribution, on the basis of the practices we describe in the following section and present in the analysis hereafter.^2^ This was done iteratively and by closely attending to participant orientations such that our intuitions regarding participants' demonstrable inferences about the presence of direct or indirect support of White nationalism were grounded in the data.^3^ Based on this process, we identified 16 cases of White nationalist remediation fulfilling these criteria, of which 6 illustrative cases (extracted from three interviews and a talk show) are presented in our subsequent analysis.

### Analyst positionality

As White researchers occupying multiple other identities working at Western universities, we acknowledge our antiracist commitments and privilege while interrogating the everyday practices that support White nationalism. Our interactional analytic approach (see next section) is premised on providing empirical evidence for claims, grounded in participants' demonstrated orientations to what they, and others, are doing. This means that we are accountable to our interpretations of the data and actions therein (c.f., Antaki et al., 2008). As members of human social interaction ourselves, we draw on our interpretive resources, within the tools attested in extant literature, to describe how participants *demonstrate* their interpretations of each other through turn‐design, recipiency and so forth. Our analysis is therefore, by design, made to make available the materials participants *and analysts* draw on in their respective conduct such that it is open to scrutiny and potential contestation.

### Data analysis

Transcripts use Jeffersonian notation (see Jefferson, 2004) and refer to individuals using their initials and interviewers using ‘IR’. We used both CA with MCA to examine, respectively, how social actions are accomplished through sequential organization, action formation and participants' orientations (Schegloff, 2007a); and making visible how categories and their associated predicates are packaged (Eglin & Hester, 1992; Hester & Eglin, 1997) including rights, obligations, knowledge, attributes and competencies.

MCA is closely related to CA in their shared analysis of talk and embodied conduct (see, Burdelski & Fukuda, 2019; Mondada, 2020). This combined framework, with its focus on how participants *do* culture, is well suited for interrogating hate speech and discriminatory conduct. Other combined CA/MCA studies have shown that racial common‐sense and moral reasoning are mobilized in interaction to perform or contest social actions (e.g. Joyce & Sterphone, 2022; Robles & Shrikant, 2021; Robles & Xie, 2024; Shrikant & Sambaraju, 2021, 2023; Waring & Tadic, 2024). Although such talk is often covert or defeasible, CA and MCA together demonstrate how racism becomes intelligible in everyday practices such as categorisation, person reference, accounts and storytelling, preference organization, and repair and self‐correction (e.g. Bolden et al., 2022; Robles, 2015; Wetherell, 2003; Whitehead, 2015, 2019; Whitehead & Lerner, 2009).

We used CA to identify and describe the sequential position and composition of actions, and MCA to explicate how categories and moral predicates were invoked to make those actions accountable (see Stokoe, 2012).^4^ Analysis proceeded iteratively: candidate sequences were identified by a multidisciplinary team of authors working in US and UK higher education, with subsequent analytic refinement through data sessions. The results, highlighted by selective cases analysed in the following sections, demonstrate participants' orientations to being in or out of the category of White nationalism and the practices they use to accomplish this management.

## RESULTS

### Self‐identified white nationalists

Here, we examine practices deployed by in or ex situ self‐identified White nationalists (or some proximate category, like Nazi/national socialist, White supremacist, etc.) towards the activity of remediating the category to which they are understood to belong in the context of the interview. That is, they recognizably protect a category treated as morally questionable, often by virtue of its connection to an action understood to be done by members of that category. Thus, they are defensive practices that respond to reproach or head off potential criticism (c.f., Ferraz de Almeida, 2022; Maynard, 2013) associated with being a member of that category.

Our first case tracks an interview between avowed White nationalist and alt‐right shock jock Christopher Cantwell (CC) and VICE journalist Ellie Reeves (IR) conducted the evening following the ‘Unite the Right’ rally in Charlottesville, Virginia, USA, in 2017. The event is well‐known for the march of predominantly men in polo shirts and slacks carrying tiki torches and chanting ‘Jews will not replace us’ (Miller‐Idriss, 2020, p. 63). This interview is framed as an interview with a White nationalist regarding a protest on behalf of White nationalism and which Cantwell treats throughout as an opportunity to promote and defend White nationalism as a project.^5^ During the event discussed, protestor James Alex Fields Jr. drove a vehicle into a crowd of counter‐protestors, injuring 35 people and killing one. In this segment, we analyse how Cantwell presents himself and his categorial co‐members as ‘reasonable’ and resultantly his categorial cross‐members as *un*reasonable.

### Case 1: Recording_3_Interview_I'd_say_it_was_worth_it


01 CC: I'd say it was worth it we knew that we were going to02     meet a lot of resistance uhh the fact that nobody on our03     side died I’d s- go ahead and call that (.) uh- points04     for us (.) the fact that (.) none of our people killed05     anybody unjustly I think is a plus for us erm and I think06     that we showed (.) err we showed our rivals that we won't07     be cowed.08 IR: But the car that struck a protestor that's un- (.)09     unprovoked.10 CC: That's <not true> >and you know that it's not true<11     you've seen the video? So.=12 IR: =I’ve seen a video=13 CC: =ye[ah  ]14 IR:    [I do]n't know much about it15 CC: um I-I-I understand [that you’re          ]16 IR:                     [(The/Tho-) CAN YOU DE]SCRIBE17     what [the vi]deo appears to show=18 CC:      [well  ]19 CC: =okay so the video appears to show someone striking that20     vehicle when these animals attack him again and he21     saw no way to get away from them except to hit the22     gas uhh and sadly (.) e-because our rivals are a bunch23     of stupid animals who don't pay attention er they24     couldn't just get out of the way of his ca:r and25     some- >and some< people got hurt and that's unfortunate



Cantwell accounts for the driver's actions based on a distinction between just and unjust killing (L04–05). This hinges on partitioning between ‘our people’ (L04)/‘our side’ (L02–3) and ‘our rivals’ (L06). This partitioning renders the killing as a reasonable consequence of being in opposition. Cantwell's attribution of ‘points for us’ (L03) and a ‘plus for us’ (L05) treats the opposition (the antiracist protestors) as an equivalent category: the predicates of both categories include killing or being killed as if in a competition wherein points may be won or lost. The categories (our reasonable White nationalist‐supporting co‐members versus antiracist/left‐leaning counter‐protestor ‘animals’) are not incidental to this justification, but provide a key logic for understanding who should be considered to have the moral highground, and as part of the ongoing narrative of White nationalism as a legitimate position.

Cantwell's response to the interviewer's challenge that the killing was not reasonable but rather unprovoked (L08–09) shifts the basis for his claim of reasonableness (L10–11, 18–25). Rather than continue with an equivalent categorisation, Cantwell dehumanizes the antiracist protestors, e.g. ‘these animals’ (L20), ‘stupid animals’ (L23), and depicts their actions as an ‘attack’ (L20), a negatively valenced activity that is bound to their dehumanized category (Robles, 2015; Tileagă, 2007; Verkuyten, 2001; Zhang, 2022). This transition from equivalent categorisation (L01–08) to negatively valenced activity bound to a dehumanizing categorisation portrays his ‘side’ as reasonable against their rivals (L22): the fundamentally unreasonable antiracist protestors. Cantwell's partitioning provides for associating a negatively valenced activity in this way: it produces an environment wherein ‘hitting the gas’ (L21–22) can be presented as the ultimate and reasonable option. Thus, blame is attributed (and attributable) to the antiracist protestors for being too ‘stupid’ (L23) to ‘get out of the way’. These partitioned categories, side‐by‐side, leverage two ways of making claims: (1) that if it is an equal competition, then either side must do what it can to ‘win’; and (2) if one side errs in a way that gives advantage to the other, then they deserve what they get—something of an ‘all's fair’ position in service of moral exculpation. These categories also promote the ongoing supportive commentary Cantwell mobilizes on the part of White nationalism, serving as an exemplar of ‘we’/‘our side’ (the White nationalists) who are bound to meet resistance (e.g. L01–02) but who would never ‘kill’ ‘unjustly’ (L04–05). This partitioning of good and bad ‘versions’ of categories, alongside allocating blame and accountability (Pomerantz, 1978; Potter et al., 1993), doing being reasonable/common‐sensical, dehumanizing and blaming accomplishes supporting White nationalism.

The next case features an interview between Matthew Heimbach (MH), co‐founder of the far‐right *Traditionalist Worker Party*, and P.J. Tobia (IR) on PBS NewsHour, a US daily news show. As with the previous case, Heimbach is not just coincidentally a self‐identified White nationalist, but is interviewed *as* representing that category and in relation to an event in which White nationalists have been associated with the death of a (counter)protestor at the rally. They are discussing the self‐same vehicular killing. Heimbach similarly constructs the driver's actions as reasonable and establishes a categorial supremacy of White nationalists over ‘mobs of the leftists’ (L06–07) to further cast violence as warranted and justified.

### Case 2: Recording_1_Interview_You're_calling_it_an_accident


01 IR: .h you'; re call- I’m s- you’re calling it an accident but the02     polic:e ar-: saying it's (.) they’r- they're charging it with03     a cri:me?04 MH:°Mhmm°05 IR: they're charging the driver with a cri:me,06 MH: Well- (.) it's a crazy situation where you've got mo:bs of07     leftists that had been busting out windows have been slashing08     tires they've been attacking people in the streets: (.) we09     don't exactly know what happened but what I do know is if10     you're in the car you're <surrou:nded> by people that are11     flat-out chanting that they want to kill you cause their12     definition of a Nazi is anyone they politically disagree with13     that is a TERrifying situation to be in especially by yourself14     so=(extract continues in case 3)



Heimbach is responding to a context in which the interviewer summarized two competing descriptions of the event: ‘an accident’ (L01) and ‘a crime’ (L03). Heimbach's ensuing account (L06–14) is well‐prefaced, signalling its non‐straightforward character (Heritage, 2015; Schegloff & Lerner, 2009). Instead of responding to the implication that his co‐members are mischaracterising events, MH works to remediate his categorial co‐member's actions in the face of an unreasonable, ‘crazy’ (L06), ‘terrifying’ (L13) situation.

As in Case 1, Heimbach works to remediate the driver's category (White nationalist) through: partitioning, disclaiming knowledge and proposing hypotheticality, and common‐sense reasoning. He first partitions the actors‐on‐the‐scene into categorial co‐members on one hand and ‘mo:bs of leftists’ (L06) on the other, to whom he binds the three‐part list of negatively valenced activities—'busting out windows’, ‘slashing tires’, ‘attacking people’ (L07–08). The ‘mobs of leftists’ make relevant possible opposing parties in an American political standard relational pair (cite) in which far‐right is contrasted with far‐left, invoking Heimbach's White nationalist category but formulating it with generic‐you (L10–11) that promotes it as the more normal or acceptable. Heimbach's subsequent disclaimer of available epistemic access (Sidnell, 2012) (L08) invites the audience to interpret conduct as a reasonable response to violent activities. To that end, he constructs a hypothetical scenario: ‘if you're in the car you're <surrou:nded> by people that […] want to kill you’ (L09–11). Third, he draws on common‐sense reasoning (Edwards, 2003), inviting the general ‘you’ to interpret the driver's action as reasonably escaping (not unreasonably murdering). MH's turn parallels Cantwell's: the killing was a consequence of a ‘reasonable course of action’ that anyone would take in a ‘terrifying situation’ (L13).

Case 3 continues with the interviewer changing his approach to challenging.

### Case 3: Recording_1_Interview_There_were_actual_swastikas_there_though


15 IR: =the↑re were actual swastikas there though which is (0.2) which16     sort of- helps meet the definition of Nazism.17 MH: there were a lot of different folks there from a lot of18     different >organisations< (.) there were constitutional19     conservatives ther- were libertarians there (.) there were20     national socialists such as ourselves there were southern21     nationalists >there were-< (.) Confederate heritage defenders22     (.) we were coming together to sa:y that we are going to stand23     together for our heritage but the left wanted to attack all of24     us (.) they want to kill anyone they disagree with (.) they25     believe in no: platform for people they disagree with and we26     use violence to get that (.) we saw the violence of the left27     yesterday and we saw also our reso:lve to defend ourselves and28     carry forward our message of defence of our faith family and29     folk.



The interviewer questions MH's use of ‘Nazi’ (L12), to which Heimbach responds through particularisation via partitioning, extreme case formulation, and puzzle‐solution format, to reorganize the categorisation of White nationalists in relation to Nazis and ‘the left’. ‘Nazi’ (Case 2, L12) surfaces as a hypothetical categorisation to which Heimbach's co‐members could belong according to the ‘mo:bs of leftists’ (Case 2, L6). The interviewer questions Heimbach's attempt to problematise its use, as ‘anyone [the left] disagree with’ (L12) by pointing to the presence of category‐bound features of Nazis (e.g. swastikas) at the protest (L15–16). Now partitioning his own co‐members (‘different folks’, L17), Heimbach counters the accusation that far‐right members were complicit in the killing, going on to specify exemplar categories (L18–21) grounded in recognizable American groups and invoking predicates of the sub‐groups. Thus, Heimbach mobilizes the normative expectation of diversity *within* a category, not to negate the interviewer's use of ‘Nazi’, but to reconcile the accusation. This casts it as a generalization (see O'Driscoll et al., 2016; Pino, 2021), and therefore misapplied, remediating the broader category in which he and his co‐members are situated. He subsequently redeploys this generalization against ‘the left’ (L23) via the extreme case formulation ‘wanted to attack all of us’ which upgrades the negative assessment by implying that ‘the left’ are indiscriminate in their attacks.

Heimbach accords positively valenced predicates like ‘stand together for our heritage’ (L23), ‘resolve to defend ourselves’ (L27), and ‘defence of our faith family and folk’ (L28) through this partitioning. Contra the previous diversification, this points to the underlying basis on which these groups can be considered in agreement, and continues to narrate White nationalism in a reasonable, even positive way (see, Long, 2023). The activities ‘stand together’ and ‘defend’ position their opponent as perpetrator. Simultaneously, ‘heritage’ and ‘faith, family and folk’ invite the audience to consider who (what category) the preceding ‘our’ invokes—albeit one that is decidedly hearable as racialised and specifically White—but in a specific context that has positioned whomever ‘we’ are as in‐the‐right.

Across cases, speakers worked to remediate objectionable conduct—action resulting in the death of another person—towards a purportedly more reasonable position. They invite the audience, via rhetorical strategies including partitioning and extreme case formulation, to interpret White nationalist assertions as common‐sense reasoning: not just legitimate, but perhaps moral. The violence perpetrated at the rally, and any future violence, is not what they *want* to do, but what they *have to do*, and what *anyone* would do. Through this, they invite identification and co‐categorisation from overhearing audiences, including those who might not consider themselves categorial White nationalists. Practices may vary, with overlap across cases, but share deployment towards the activity of remediating the category in question. What we show in the next section is that they are also used by non‐members of these categories who are similarly engaged in the activity of remediating them.

### Doing white identity politics

Having established some practices deployed by self‐categorized members of the category ‘White nationalist’ in support of remediating the category (often through remediating its members' actions), we now move to the actions of those who do not self‐categorize as such. These members are those who may even disclaim or contest the category or what it means, but who still participate in the activity of remediation through their various other accounts, defences, etc. As we show, these members engage in many of the same practices as self‐categorized White nationalists, thus accomplishing support of the same activity: remediating the category. Resultantly, we demonstrate how participants become situationally hearable as members of the category ‘White nationalist’, a feature of having produced a ‘dog whistle’.

Cases 4 and 5 come from a news interview between US Senator Tommy Tuberville (TT) and CNN journalist Kaitlan Collins (IR) conducted in early July 2023. Earlier in 2023, Tuberville received wide criticism for comments referring to White nationalists in the military as merely ‘Americans’ (L3). Following Tuberville's support for blocking military promotions, Collins used this interview to invite Tuberville to ‘clarify’ his prior comments about White nationalists in the military, following which audio of his comments was played.

### Case 4: Recording_8_Interview_Do_you_believe_they_should_allow_White_nationalists_in_the_military?


01 IR: Do you believe they (0.4) sh↑ould allow (.) White nationalists02     ↑in (.) the militar↑y?03 TT: well, they ↑call them that. (.) ↑I call them Americans.04     (3.0)05 IR: do you want to explain those comments senator?=06 TT: =yeah. First of all. (.) uh. I’m to::tally against a:ny type of07     racism. Okay. I was a football coach for forty years. A:nd (.)08     I dealt- and- and- had a opportunity to d- to be around .hh more09     minorities than >anybody< up around this HIll. .hh but when OUR10     military iz bein’ attacked, was bein’ attacked (.) after ni:↑:ne11     eleven. u- after January the sixth. and that was my first day on12     the senate floor. I thought it was outrageous (.) of what (.)13     senators from the democratic si:de (.) Chuck Schumer w- said on14     the floor that night (.) calling out people, calling people racist,15     calling people nationalist=White nationalist,=↑White nationalist is16     just another word that they wanna use .h other than racism. .hh uh:17     ((shakes head)) (1.5) I’m TOtally against anything to do with racism18     (.) but the thing about being a White nationalist is just a (.)19     ↑cover word for the democrats now where they can use it to try20     make (.) people ma:d across the country, identity politics, .hh21     I’m totally against that. But I’m for the American people, I’m22     for military, I’m for Christian Conservatives (0.4) Democrats23     who↑ever wants to be in the dem- uh- the- the military. To fight24     For this country t’ protect this country. That's what it's all about.25 IR: But j'st to be clear. <You agree> (0.8) that White nationalists26     should not be serving in the U.S. military. Is that what you’re27     saying?28 TT: If- if ↑people think that a White nationalist is a racist, (1.0) I29     agree with that (.) I w’d agree that th[ey sh’ldn’t-]30 IR:                                        [a  White  na]tionalist is31     someone who believes that (.) w:- the White race is superior to32     other races33 TT: *Well tha- that's some people's opinion. Uh and ↑I don’t think34     *((shaking head))35     [I mean- a lot        ] uh-36 IR: [that's not an opinion]37     (0.8)



Tuberville's reaffirmation of his prior comments orients to a possibly duplicitous question design through well‐prefacing and the ambiguous referent ‘they’ (L03) who are using the term ‘White nationalists’ compared to Tuberville's ‘Americans’. Following a prompt to elaborate, Tuberville disavows the racist implication of his comments, demonstrating his orientation to such an available hearing—‘to::tally against a:ny type of racism. Okay’ (L06)—with prosodic stress and later repetitions (L17, 21). He further proposes his epistemic status and thus rights to speak compared to others based on his category membership ‘football coach for 40 years’ (L07) (a form of category entitlement, c.f., Wiggins, 2017; see also Coupland, 2003 on historicity), and experience around ‘more minorities than anybody up around this hill [colloquialism for US congress]’ (L09). This assertion unpacks some (and obscures others) of the inferences that could be made in this (US) context in which categories are set with and against each other; for example, the history of minorities in American football, the contrast between elite over‐educated ‘career’ politicians and contact with minorities, and the association between elite politicians and representatives of the Democratic party.

In proposing his rights to speak as a moral and epistemic authority (Stivers et al., 2011)—namely, as someone who has ‘had the opportunity to be around more minorities’ because playing football is proposedly bound to the category minority—he is setting up an increasingly specific contrast: first to ‘them’, then ‘anybody up around this hill’, and, ultimately, to ‘senators from the democratic si:de’ and ‘Chuck Schumer’ (L13). Indeed, it is ‘they’ who were not supporting—or even attacking—the military and ‘try[ing] to make people mad’ (L19–20). Tuberville's positioning of Democratic Senators as his opponents is made starker by his list of US categories with which he is aligned: ‘the American people’, ‘military’, ‘Christian Conservatives’ (L21–2). Thus, he establishes a partition between himself and his categorial co‐members, on the one hand, and the Democratic Senators, on the other, such that the latter are associated with antagonistic actions and the former with supporting Americans, the military, and opposing racism.

Simultaneously, Tuberville engages in another partitioning project: one that particularizes the category ‘White nationalist’ such that it is categorically separate from ‘racists’ (L15–19). Indeed, his use of ‘White nationalist’ provides for being a member of both ‘White nationalist’ and ‘racist’ (L28), but rejects the two as coextensive. Rather, as becomes evident (Cast 5, L63–64), Tuberville seemingly deploys ‘White nationalist’ as a compound category, such that it refers to nationalists who are White (a category with a particular history in the US). In doing so, he problematises IR's proposed partition, whereby members of ‘White nationalist’ are *necessarily* racists, whether as a category‐bound activity or as co‐extensive categories. Tuberville's particularisation suggests that *some* White nationalists are racist, but not all—an anti‐generalization strategy comparable to Heimbach's in Case 3, which contests the category overlap presumed by interview questions.

Despite Tuberville resisting taking a stance on White nationalists using both partitioning strategies, IR pursues getting Tuberville on the record using a polar interrogative (Koshik, 2002): ‘You agree that White nationalists should not be serving in the US military. Is that what you're saying?’ (L25–27). Like in Case 2, Tuberville relies on hypotheticality to present a reasonable situation, but unlike Heimbach, Tuberville is deploying a hypothetical to present himself as reasonable in the face of an unreasonable (or problematic) question (see Raymond, 2003 on nonconforming responses to polar questions). In doing so, he reaffirms his prior partitioning by hypothetically agreeing that *racists* should not be in the military, but only conditionally agreeing to the interviewer's framing that *White nationalists* should not be. Posing White‐nationalists‐as‐racists as a hypothetical allows him to avoid taking a stance of his own on that issue, instead treating it as mere opinion (L33), thus resisting providing an answer to IR's question while nonetheless progressing the interaction (Joyce, 2022).

Thus far, Tuberville's responses have been partly organized around evading being hearable as a member of the category White nationalist, and specifically White nationalist *qua* racist, while doing targeted work to remediate the moral standing of the category. It is, in part, through this that Tuberville opens himself up to being hearable as a White nationalist—i.e., through preempting disclaiming racism, partitioning, particularisation (against generalizing White nationalists as racist), disclaiming stance via hypotheticality, remediating the category, and attacking those who negatively assess White nationalists. IR's subsequent repair initiation produced in overlap and rejecting Tuberville's epistemic manoeuvre (L36), demonstrates their orientation to Tuberville's problematic position. His response, and the encounter, continues in Case 5.

### Case 5: Recording_8_Interview_What's_your_opinion?


38 TT:  Pardon?39 IR:  What's your opinion?40 TT:  My opinion of a White nationalist-=if somebody wants to call ‘em a41      White nationalist_t’ me is an American. It's an American. No:w if42      that- (.) White nationalist is a racist? I’m totally against anything43      that they wanna do. Because I am one hundred and ten percent against44      racism. But I want somebody (.) that's in our military, that's45      strong, that believes in this country, (.) that's an American (.)46      that will fi:ght (.) along a:nybody whether it's a man or a woman,47      Black or White, re(-/d), it doesn’t make any difference .hhh uh:: an-48      an so I’m a to:tally against identity politics. I think it's ruining49      this country, and I think the Democrats oughtta be ashamed for how50      they’re doin’ this, because it's divi:ding this country and its51      making this country s:kweaker >every<day.52 IR:  B’t that- that's not identity politics. You said a White nationalist53      is an A[merican ]54 TT:         [it ↑is i]dentity politi:cs=55 IR:  =you said a White nationalist is an American but a White nationalist56      is someone who- (.) who believes horrific things, you don’t, do you57      really think that's someone who should be ser↑ving (.) in the58      military?59 TT:  Well that's just a name that's been given. I mean-60      [I- l- listen61 IR:  [↑it's not it's a rea]l it's a real definition.62      There's [real concerns about extremism.    ]63 TT:          [s- s- so if you’re gonna do away w]ith m- most White64      people in this country outta the military we got huge problems.65 IR:  It's not [w- it's not (.) w- it's not people who are      Whi]te.66 TT:           [we got huge problems. We’ve got ↑huge problems. Hu-]67 IR:  It's White nationalists.68 TT:  That have a few pro[bably different beliefs.]69 IR:                     [You see the distinction r]ight?]70 TT:  That have- that have different beliefs. N↑ow (.) if racism? is one71      those beliefs? I’m totally against it. I am totally against racism.72      .hh [But there's a lot of people who believe in different things]73 IR:      [But th- that is- that is a White- a White nationalist]74      is racist senator75 TT:  Well th- (.) th- that's ↑your opinion. That's [↑yo]ur opinion76 IR:                                               [it-]77 TT:  [But if it's racism  ] if it's racism I’m totally against it. I’m78 IR:  [It's not an opinion]79 TT:  totally against any type of racism. Or- any any type of racism I80      don’t care what it's in ((video ends))



When Tuberville ultimately gives ‘[his] opinion of a White nationalist’ (L40), he affirms candidate hearings of the earlier partitioning whereby “White nationalists” and ‘racists’ are distinct insofar as ‘White nationalists’ belong with ‘American’ (L41). Beyond that, he also introduces a hypothetical scenario: instead of ascribing categories and predicates, Tuberville treats the category as other‐ascribed and therefore not necessarily one he would use (L40–42). Additionally, he preemptively positions himself as invariant to all exigencies: ‘I am one hundred and ten percent against racism’ (L43–44) (see Pomerantz, 1986, on extreme case formulations). This trajectory—particularisation followed by hypotheticality and preemptive defence—plus accusations of insincere politics, renders his position defeasible; possible challenges are weakened because Tuberville claims category misapplication, allowing him to retreat to an alternative position. This continues across the remainder of the encounter, with IR attempting to solicit an admission that White nationalists are racist (L52–53, 55–58, 61–62, 69, 73–74) and Tuberville evading each attempt.

Claims of not being racist, category particularisation via partitioning and presenting other‐ascribed categorisations as hypotheticals form part of the basis for Tuberville's activity being legible as dog whistling. These are not coded messages that invite the audience to fill in with common‐sense reasoning (although these may be present as in Case 3), but occasioned rhetorical strategies that bind ‘White nationalist’ to positively valenced categories and predicates like ‘American’ (L41) and being ‘against racism’ (L71). This intersects with the ambiguity stemming from the hearability of Tuberville possibly regarding ‘White nationalist’ as a compound category. As such, Tuberville can treat proposals like removing White nationalists from the military as being about removing all White people from the military (L63–64) or targeting people ‘who believe in different things’ (L72).

Finally, Tuberville's possible hearable proximity to the category White nationalist comes not only from his defence of it, but also from his attacks on its critics. Specifically, he targets the ‘Democrats’ (L49) for their use of multiple categories as a basis for accusing them of ‘dividing this country’ (L50). This accusation, alongside ones of using White nationalism as merely another levy of racism (L15–16) and engaging in ‘identity politics’ (L19–20, 48–51), helps to build a broader allegation of ‘insincere’ politics, that is, politics that claim to be ‘doing’ one thing but are, in fact, ‘doing’ another (see Goffman, 1959, 1970, on ‘insincere’ actions and talk). This mirrors Heimbach's approach in Case 3: an accusation of insincerity targeting partitioned out‐group members (‘the left’, ‘Democrats’) that remediates the category White nationalist by absolving it of the attributed wrongdoing since the accusation was insincere. Through this parallelism, Tuberville may be conceived as hearable as Heimbach's co‐member (i.e., a White nationalist). This potential hearability is visible as a members' orientation in IR's repeated attempts to pin Tuberville down to a position critical of White nationalists, as seen in her question design (e.g. ‘You see the distinction, right?’, L69, also L55–58, 61–21) that treats his position as not ambiguous, but, again, potentially hearably White nationalist‐aligned or ‐supportive.

Case 6 comes from GB News, an opinion‐oriented UK‐based television channel. The encounter took place in May 2023 in front of a live audience (AUD). Prior to the excerpted segment, the panellists—Leo Kearse (LK), Cressida Wetton (CW), and Francis Foster (FF)—watched and commented on a video of then‐US president Joe Biden making a speech against White supremacy to an audience of graduates at Morehouse College, an (Historically Black College and University). The ensuing commentary targets ways that Biden's speech is hearable as insincere (Goffman, 1959, 1970), specifically in his assessment of White supremacy as a ‘poison’ and ‘the most dangerous terrorist threat to our homeland’.

### Case 6: Recording_21_Talk Show_I_must_admit_I_don’t_know_the_context


54     CW:   Yeah, (.) uh, [I m(h)e- w(h)uh- I do- heh- I must admit, I55     AUD:                [((laughter))56     CW:   don’t know the context, um, but (what I-[ sa:w)57     LK:                                        [well it's probably58           White supremacy which is actually quite a[n- (.) quite an59     CW:                                            [(wellificould-)60     LK: interesting thing, because, ehh, we’ve seen a lot of White61           supremacist- t- (.) White supremacy recently in Ame:rica62           that isn’t by: White people, >so theres<, ehh,63           >whu was he called<, ehh, Mauritzio, ehh:, Garcia? Ehm, (.)64           (((pointing at FF))°r-[ related to your mum°=65     CW: [How inclusive!66     FF: =[is he? Heh heh heh heh67     LK: [no- um, who- ehh, whos, >e- u-< shot- shot up a MALL in-68           in TEXas a:nd is been (.) l:abelled a White supremacist-69           but he's actually Mexican, which- y’know I- I think a lot70           of White supremacists would have some (.) ISSue with. They71           tend to be (.) ehh quite specific about the- about ~r- r-~72           race,=73     ???:  =(↑hmm/(chuckle))74     LK: An:d, and [ther- there also- the leader of the ↑Proud Boys74     FF:             [↑th- th-75     LK:   is eh:, is a black Cuban guy.=76     FF:   =Yeah it i- look, the quality of White supremacy s just77           gotten do:wn over the years.78     ~FF:  ((laughter))79     FF:   >Djeknow what I mean? ↑Djuremember the Nazis? >Say what you80           like about ‘em<, those boys were organised, they looked81           great.82     ~FF:  ((chuckles))83     FF:   Y’look at these ones no:w, mate, rubbish!84     CW:   ((chuckles))85     LK:   And that's the bit we’re gonna clip today-86     ALL:  ((laughter))



After undermining Biden's talk by reproducing as laughable through sarcasm (see, Norrick, 1994; Pexman & Olineck, 2002), the participants turn to discuss Biden's ‘White supremacists’, also taking up their potential hearability as co‐members therewith. In doing so, they make use of (both in‐ and out‐group, and particularizing) partitioning and ventriloquising White supremacists to produce laughables. LK shifts the conversation from Biden towards a general reflection on White supremacy and its modern characteristics, noting the ‘interesting’ phenomenon of White supremacy being claimed by people identified as not White. The marked character of the observation provides initial evidence of LK's orientation to a discrepancy between category and action—that is, between non‐White perpetrator and *doing* White supremacy (c.f., Edwards, 2003). Further evidence comes in how LK identifies the alleged White supremacists: first with a (Spanish) name alone, followed by someone categorized as ‘actually Mexican’ (L60–63, 69) and then an unnamed ‘Black Cuban guy’ (L75). The actions attributed to each are presented as laughables due to their discrepant nature (Okazawa, 2021), with the escalating list of three ending with ‘the leader of the Proud Boys’, a far‐right, neo‐fascist organization. LK also ascribes the action ‘labelled’ to their candidate membership in the category ‘White supremacist’, underscoring the implicit ‘interesting’ partition between ‘real’, White ‘White supremacists’ and ‘alleged’ non‐White ‘White supremacists’. This implicit line of joking becomes explicit with FF's reference the Nazis having ‘looked great’ (L79–81) and ‘these ones now’ being ‘rubbish’ (L83), a trajectory that receives supportive laughter from the panel.

This joke relies on animating (Goffman, 1981), i.e., voicing without initially composing or necessarily believing, the position of a White supremacist who has ‘issues’ with non‐White perpetrators being placed in the category White (L69–72). In addition to the component complimenting Nazis (L79–81) and denigrating so‐called modern, non‐White Nazis (L83), FF states ‘the quality of White supremacy's just gotten down over the years’ (L76–77), all interspersed with laughter. LK concludes the sequence with a joking remark that orients to the hearable offensiveness of the preceding remarks ‘out of context’ (L85). In doing so, he treats FF's comments as examples of institutionally appropriate, nonserious content, not attributable to FF personally—they are framed as insincere and for comedic purposes. To hold FF accountable—as supportive of Nazis, as himself a White supremacist, etc.—would thus be, per LK, a mishearing on the recipient's part.

By joking about the seriousness of White supremacy and White supremacist violence, the commentators contribute to the project of the far‐right. Animating the views of White supremacists positions them as comfortable ‘playing’ the category or as even possibly hearable as co‐members in the category, a possible outcome to which they orient. Compare this to the derision with which political cross‐members were mocked (see, Robles & Xiong, 2024) for associating with the category ‘Black people’. Thus, they asymmetrically talk these racial categories into being.

## DISCUSSION

Our findings demonstrate how self‐avowed White nationalists may subvert otherwise negatively valenced categories in the mainstream, and how others who may publicly resist or presume themselves to be outside of this category may nonetheless support and normalize White nationalism using many of the same practices. We thus demonstrate that actions supporting White nationalism can be accomplished in many ways with or without explicit self‐identification with the category, producing the effect that this form of racism is somehow felt widely—but rarely explicitly seen.

We have described a number of practices participants use to manage their relationship to the category of White nationalists—whether they avow or avoid it. These emerge as responses to being held accountable for some prior talk or event that is being associated with racist and White nationalist viewpoints. Attempts to justify or distance themselves from membership in the White nationalist category include partitioning, extreme case formulations, puzzles, hypotheticality, attributions of insincerity, producing laughables, and accusations. Many of these practices occurred systematically across our dataset of 16 cases and are highlighted in the six excerpts presented herein; for example, partitioning was a recurrent practice for separating negative category attributes (unthinking violence, racism, etc.) from White nationalism by separating out some other or opposed category. Other practices were more situation‐specific, for example, the laughables of the talk show setting in case 6, like other such examples in our data, reflect the entertainment function of these settings rather than the more serious political interviews (as with the US senator). Furthermore, more explicit discussion of politics that invoked party membership easily produced orientations to the insincerity of other parties and their accusations of racism.

While slight variations exist in accordance with a particular setting or interactional environment, put together, all these practices were marshalled to protect (and potentially normalize) White nationalism while deflecting accountability for the racism of White nationalist ideologies. This relationship between what is overtly communicated in the interactional moment versus what is covertly signalled to potential overhearing audiences is part of what constitutes these practices as ways of doing dog whistling.

Members of society have been shown to use race and racism to make sense of conduct and culpability (e.g., Johnson et al., 2024; Shrikant & Sambaraju, 2021), and, in light of this, actors have developed a range of practices for circumventing attributions of racism (e.g., Billig, 1988; Billig et al., 1988; Quaranto, 2022; Whitehead, 2020). Indeed, members do work to not be hearable as prejudiced or racist, ranging from on‐record denials, which may themselves do violence (Lentin, 2021), to, for example, setting up puzzles that invite the hearer to provide a category or action as the solution (e.g., Whitehead, 2009), as seen in our data. Examining this interactional work has been central to our approach and revealed how actors remediate the category ‘White nationalist’ when it is associated with ‘racism’ and/or (racist) ‘violence’.

Our analysis described practices participants use to provide cover for White nationalism ideologies, whether they would claim the category or not. These data draw on mediated, often interview interactions, which is a particular genre with certain conventions and expected audiences. While this may limit claims about other contexts, it is well suited to examine how White nationalism is signalled to general audiences, and how interviews and similar settings create opportunities for generating certain recipiencies with large‐scale (mass‐mediated) effects (c.f., Clayman, 2004; Robles & Xie, 2024). Talk in interviews, political debates, panel shows, etc. is produced for both co‐present interlocutors—e.g., interviewer, moderator, co‐panellists, etc. and for an overhearing audience (possibly co‐present in the studio, and certainly over‐the‐air). As such, speakers design turns under multiple institutional constraints and discursive recipiencies, including adequately answering institutionally constrained questions while also attending to categorial and political concerns of constituents—i.e., the *content* of the question (e.g., Case 5 L55–74). Furthermore, while this selection from our corpus is but a snapshot of particular moments in English‐speaking media, we propose these practices could be observable in many languages and settings where people avoid being held accountable for violent ideologies.

These practices rely on norms governing who may challenge whom, and on the presumption of a potentially supportive base that will ‘read’ their conduct as it is intended. This is true both in general and of specific practices. Consider accusations of insincerity, which trade on the idea that what one claims to be doing and what they are in fact doing are not the same. When Tuberville says Democrats are accusing people of being White nationalists, he suggests that they do not literally mean ‘White nationalists’ but use it for ulterior political motives. So a secondary argument for the overhearing audience is that attributions of racism—smuggled here as ‘White nationalist’—are inherently politically invested and not to be taken seriously, in part because racism itself is not to be taken seriously.

Dog whistling more broadly requires a relationship between what is overtly stated and what is covertly communicated and recognized. Dog whistles rely on the turn‐design work of the speaker—i.e., how they orient to recipiency and (multiple) possible audience(s)—*and* the recipient(s) interpretive work making sense of, or ‘hearing’, that which is covertly ‘whistled’. These practices depend on a potentially supportive base, whether categorial White nationalists or those who might disclaim the category if made explicit. This happens through invitations to hear oneself in broad categories like ‘us’, ‘we’, and ‘our’, making use of the *knowledge protected against induction* (Sacks, 1992: 336) carried in categories. By ratifying the views and rationalizations of White nationalism through laughter, silence, unchallenged sharing, and justifying/displaying understanding, participants can become complicit in the project of White supremacy. It does not require a categorial White nationalist to promote White nationalism.

## CONCLUSION

White nationalism is not merely one more political belief among many, but one that promotes the differential distribution of rights and privileges, supports violence, and does so through the propagation of a race realist formulation ‘racial common‐sense’. We contend that the unambiguous violence referred to and justified by the speakers in our first cases—the hate crime and first‐degree murder by vehicle—is supported by the symbolic violence inherent in normalizing and/or laughing off White nationalism featured in our dataset. We examined *how* this was accomplished, demonstrating the shared practices used by those who self‐identify as White nationalists and those hearable as such in the unfolding interaction. This work advances the value of discursive and interactional approaches to social injustice (Shrikant et al., 2022) by building on what has already been shown while advancing further into the more challenging areas of covert racism which require more empirical investigation.

These results support work on how bigotry can be undermined and challenged (see, e.g., Joyce & Sterphone, 2022) by making visible how practices used by White nationalists exculpate racist views. Researchers can highlight such practices (especially where covert) such that they can be more readily challenged; and this can feed into work that makes such visibility and challenging more accessible generally (e.g., DARG, n.d.; Xie, 2025). Through this work, we have come to illuminate ‘the kinds of things they work with’ (Sacks, 1987, p. 67), and thus, the specific resources deployed in support of those projects. We return to Rawls et al. (2020) keen insights on the political value of ethnomethodological and conversation analytic research agendas: ‘if we wish to bring about change, we will need to know what we are up against’ (p. 26). Focused study of the practices that reproduce and propagate White nationalism provides resources for understanding where and how challenges might be produced and structured.

## AUTHOR CONTRIBUTIONS

**J. Sterphone:** Conceptualization; investigation; writing – original draft; methodology; validation; writing – review and editing; formal analysis; project administration; supervision; data curation; funding acquisition. **Jessica Robles:** Conceptualization; writing – original draft; methodology; validation; writing – review and editing; formal analysis; investigation. **Jack B. Joyce:** Conceptualization; investigation; writing – original draft; funding acquisition; methodology; validation; writing – review and editing; formal analysis; data curation; resources. **Natalie Flint:** Investigation; writing – original draft; writing – review and editing; validation; methodology; visualization; formal analysis; data curation. **M. J. Hill:** Conceptualization; writing – original draft; investigation; methodology. **Q. A. Ellis:** Investigation; data curation; writing – original draft.

## CONFLICT OF INTEREST STATEMENT

The authors declare no conflict of interest, financial or otherwise, with respect to the research, authorship, or publication of this article.

## Data Availability

The data that support the findings of this study are available from the corresponding author upon reasonable request.

## Footnotes

^1^
Note that our contexts and the work we build on joins a tradition of examining the racist speech of White elites—such talk might look different in areas across groups that do not involve majority White populations (c.f., Sambaraju, 2025).

^2^
We did not categorize participants as White or as White nationalists from an analyst's perspective; rather, we examined contexts where White nationalists (regardless of race presentation) were treated and discussed *as* White nationalists *or* where participants were questioned or discussed (or potentially questionable as) White nationalists due to some public/mediated action.

^3^
The relatively large research team, alongside iterative data sessions and ongoing engagement with relevant literature, supported the development of distinctions between explicit White nationalism (participants' self‐identification), implicit White nationalism based on other‐attribution (making accounting for that attribution relevant), and implicit White nationalism based on participant orientations to the possibility of an attribution of White nationalism. These analytic distinctions were grounded in participants' actions and displayed recognitions of accountability for interpretably white nationalistic content of their talk. Analytic claims were based on participants' interactional orientations rather than categories derived from analysts' assumptions about participants' apparent Whiteness or White‐passing status (c.f., Tam & Rafaely, 2025).

^4^
This also included some consistent concepts from discursive psychology (Wiggins, 2017) which is cited where particular to that work; however, I have typically accessed these materials through MCA/CA (while acknowledging the relationship between these approaches is complex).

^5^
Due to the institutional features of the interview, we treat the interviewees in Cases 1–3 as omnirelevantly potentially White nationalists. That is, we do not treat them as at all times White nationalists, but as at all times potentially relevantly categorizable as such due to the fact that they are being interviewed precisely for their possible categorisation as such.

## APPENDIX A TABLE OF DATA

**TABLE A1:** Data collection summary—all data are video except where indicated by an asterisk* and all data are publicly available.

Data ID	Genre	Description	Length	Date collected
Recording 1	Interview	Charlottesville Protest (post‐protest). Interview broadcast on PBS. Charlottesville Protest (pre‐protest)	1m10s	25/03/2021
Recording 2	Interview	Charlottesville Protest (pre‐protest). Christopher Cantwell interview with Elle Reeve for Vice News. Charlottesville Protest (post‐protest)	1m25s	25/03/2021
Recording 3	Interview	Charlottesville Protest (post‐protest). Christopher Cantwell interview with Elle Reeve for Vice NewsCharlottesville Protest (post‐protest)	2m28s	25/03/2021
Recording 4*	Interview	Follow‐up interviews on politicians' comments on White nationalists in the US military	10m13s	16/12/2023
Recording 5	Interview	Vivek Ramaswamy interview on CNN talking about the death of three people after a racist shooting in Jacksonville. Vivek Ramaswamy CNN	10m51s	16/12/2023
Recording 6	Interview	NBC coverage of Senator Tuberville's racially charged comments and broadcast of Tuberville's speech and panel discussion. Tuberville	2m02s	16/12/2023
Recording 7	Interview	CNN coverage of Senator Tuberville's comments about White nationalists, featuring interviews with experts. Tuberville	7m23s	16/12/2023
Recording 8	Interview	Senator Tuberville interview on CNN about his blockade of military promotions. Tuberville interview on CNN	10m57s	16/12/2023
Recording 9	Interview	GB News interview with Sir Desmond Swayne on his opposition to expanding Afghan resettlement or refugee intake, framed around his belief that the UK has reached its capacity for new arrivals. GB News	3m43s	07/02/2024
Recording 10	Parliament	German Parliament. Brandner argues for constitutionally declaring German as the national language due to immigration. German Parliament	6m18s	04/05/2023
Recording 11	Protestor	UK Protest—street debate about religious ideology, terrorism, and moral responsibility. Discussion about religious ideology, terrorism, and moral responsibility. UK Protest—Christianity/Islam	2m22s	25/03/2021
Recording 12	Protestor	US Protest – Anti‐abortion. ‘How do you know what a person is?’ US Protest—Abortion	5m54s	25/03/2021
Recording 13	Protestor	US Protest—Argumentative engagement with a protest against anti‐Muslim bigotry. US Protest—Anti‐Muslim	7m55s	25/03/2021
Recording 14	Public	Public argument on a Bbus—People that speak English passenger accuses another passenger (and her family) of not coming to the country legally	3m01s	25/03/2021
Recording 15	Public	Public argument on London Underground—later the argument becomes physical. Tube	3m02s	25/03/2021
Recording 16	Public	Public argument on London Underground—anti‐immigration ‘taking the English jobs…’. Tube	1m24s	25/03/2021
Recording 17	Public	Public argument on London Underground—passenger asks a fellow passenger to turn their music down—becomes an escalated argument. Tube—Playing music	5m30s	25/03/2021
Recording 18	Public	Public argument on New York City Subway—passenger accuses two young people of having no manners	2m56s	25/03/2021
Recording 19	Public	Public argument on London Underground—accusations of racism from both parties as both parties call each other names. Tube—‘White slag’	1m18s	25/03/2021
Recording 20	Talk Show	Real Time with Bill Maher panel discussion featuring Ben Shapiro, and Malcolm Nance. The relevant portion involves Maher talking about critical race theory in schools. He frames objections in terms of children being made to ‘fixate on race’ or feel ‘collective guilt’. Ben Shapiro	9m31s	20/11/2023
Recording 21	Talk Show	GB News's Free Speech Nation, featuring Leo Kearse, Cressida Wetton, and Francis Foster. The clip centres on a video of Joe Biden speaking at an HBCU about white supremacy as a national threat. The hosts and audience treat the clip as a springboard for satire. GB News—Biden ‘White supremacy … terrorist threat’	2m19s	07/02/2023
Recording 22	Talk Show	The New Culture Forum interview with Konstantin Kisin. Discussion that the West should reject feelings of guilt about historical slavery and racial issues. It promotes a confident, assertive view of Western identity rather than apologizing for past injustices. Konstantin Kisin	28m44s	16/12/2023
Recording 23	Talk Show	Andrew Tate and Candace Owens discuss the degradation of Western culture, the erosion of masculinity, and the influence of global elites. Daily Wire – Andrew Tate	3hr05m29s	16/12/2023
Recording 24	Talk Show	GB News discussion featuring Leo Kearse and two panellists discussing a Premier League decision to pause matches during Ramadan so Muslim players can break their fast. GB News – Referees	1m33s	04/03/2023
